# Distance to health services influences insecticide-treated net possession and use among six to 59 month-old children in Malawi

**DOI:** 10.1186/1475-2875-11-18

**Published:** 2012-01-11

**Authors:** Peter S Larson, Don P Mathanga, Carl H Campbell, Mark L Wilson

**Affiliations:** 1 Department of Epidemiology, School of Public Health, University of Michigan, 09 Observatory, Ann Arbor, MI 48109-2029, USA; 2 Malaria Alert Centre, College of Medicine, University of Malawi, Blantyre, Malawi; 3 Department of Community Health, College of Medicine, University of Malawi, Blantyre, Malawi; 4 Center for Tropical and Emerging Global Diseases, University of Georgia, Athens, GA, USA

## Abstract

**Background:**

Health ministries and providers are rapidly scaling up insecticide-treated nets (ITN) distribution to control malaria, yet possession and proper use typically remain below targeted levels. In Malawi, health facilities (HFs) are currently the principal points of ITN distribution, making it important to understand how access to these ITN sources affects ownership, possession, and use. The authors evaluated the association between proximity to HFs and ITN possession or use among Malawian children six to 59 months of age.

**Methods:**

A household malaria survey undertaken in eight districts of Malawi during 2007 was used to characterize ITN possession and use. The location of each respondent's household was geocoded as was those of Ministry of Health (MoH) HFs and other health centres. Euclidean distance from each household to the nearest HF was calculated. Patterns of net possession and use were determined through descriptive methods. The authors then analysed the significance of distance and ITN possession/use through standard statistical tests, including logistic regression.

**Results:**

Median distance to HFs was greater among households that did not possess ITNs and did not use an ITN the previous evening. Descriptive statistical methods confirmed a pattern of decreasing ITN possession and use with increasing distance from HFs. Logistic regression showed the same statistically significant association of distance to HFs, even when controlling for age and gender of the child, ratio of nets to children in household, community net possession and use, and household material wealth.

**Conclusions:**

Strategies that exclusively distribute ITNs through HFs are likely to be less effective in increasing possession and use in communities that are more distant from those health services. Health providers should look towards community-based distribution services that take ITNs directly to community members to more effectively scale up ITN possession and regular use aimed at protecting children from malaria.

## Background

Insecticide-treated mosquito nets (ITNs) have reduced all-cause childhood deaths by 15-20% according to diverse research evidence [1-7]. ITN programme evaluations have also demonstrated substantial impacts against malaria [8-10]. Greater coverage has produced enhanced reduction in malaria morbidity and mortality, even more so among those within a close proximity of households with treated nets [11]. Given these benefits, as well as the low cost, safety and ease of ITN implementation, national malaria programmes are now scaling up ITN distribution in attempting to achieve the Roll Back Malaria target of 80% coverage.

In Malawi, nearly all residents live at some level of year-round risk of *Plasmodium *infection. The Malawi Ministry of Health (MOH) estimates that about six million malaria episodes occur each year [12] among a 2010 population of 15 M people [13]. To control malaria, the Government of Malawi is rapidly scaling up effective malaria interventions [14]. Since 1998, the ITN programme in Malawi has distributed cost-subsidized ITNs to children under 5 years of age, and to pregnant women, through public Health Facilities (HFs) and short-term mass distribution campaigns. According to UNICEF's Multiple Indicator Cluster Survey (MICS) and the recent Malawi Malaria Indicator Survey (MIS), possession of at least one ITN has increased from 49.5% in 2006 to approximately 63% in 2010 with utilization for pregnant women and children under five increasing from 26% to 49% and from 23% to 59%, respectively [12,15]. However, as has been shown in past studies, possession and use can vary significantly by district [16].

Although ITNs are an effective means of preventing transmission, inequities exist both in use and distribution. [17] and [18] demonstrated that socioeconomic status (SES) was a major influence on knowledge of and access to health care, even in rural areas considered to be uniformly very poor. In countries where ITNs are partially subsidized and socially marketed, cost is the main factor which prevents the poorest of the poor from accessing and utilizing them [19,20]. To reduce barriers to possessing an ITN, starting in 2007, the Government of Malawi has been providing free ITNs to pregnant women and children < 5 years old (yo) attending a public HF [21]. Although coverage has significantly increased since the implementation of facility-based free ITN distribution, the programme remains inequitable. Coverage in urban areas is still much higher than that in rural areas [12,15].

Although many health ministries and NGOs widely distribute ITNs for free or at low cost, their incorrect and inconsistent use remains problematic. Usage patterns differ among age groups [22], house construction, sleeping configuration [23,24], education level [25]. This suggests that possession and distribution are only a first step towards reaching widespread household use of ITNs. This is particularly important for the rural poor, who often reside in areas of diminished public health infrastructure and challenging, vector-friendly topography. In addition, poor households often live in rudimentary conditions. Hence, the members of these households live under the highest risk of disease [23].

Distance to health services impacts on health seeking behaviours [26,27]. Travel times, lack of access to transportation, and seasonally inaccessible roadways can present barriers to patient access to health facilities [28]. Areas of low access are often inhabited by people who need healthcare the most [29]. Ill residents in areas where access is difficult often under-utilize services or present to health facilities (HF) only when their condition is grave, sometimes missing opportunities to effectively treat health problems [30]. Distance to HFs may also negatively impact disease prevention. For malaria in particular, remoteness and proximity to HFs have been shown to be associated with ITN possession in Kenya [31]. Proximity to health services and ITN distribution points might also influence regular and proper use of ITNs. Accordingly, the authors examined household-level determinants of reported ITN possession and use among six to 59 month-old children in Malawi, using data gathered during a 2007 survey of malaria patterns. Associations between geographic distances to HFs and reported ITN possession and use were evaluated.

## Methods

A population-based, cross-sectional survey was undertaken in eight of Malawi's 28 Districts during April/May 2007. Strategically conducted at the end of the rainy season when malaria-related morbidity is normally highest, the Districts were chosen from throughout the country. Surveys were conducted in Blantyre, Mwanza, Phalombe, and Chiradzulu Districts in the Southern Region, Lilongwe and Nkhotakhota in the Central Region, and Karonga and Rumphi in the Northern Region. The Institutional Review Boards at the College of Medicine, University of Malawi and the US Centers for Disease Control and Prevention (CDC) approved the study protocol.

### Household selection and sampling

Households were selected for inclusion in the study using a modified EPI cluster survey method as described by Turner et al. [32]. The modified cluster survey method involved: a) selection of Enumeration Areas (EAs) from each of the eight Districts with probability proportional to size (PPS) of the population b) use of EA maps to create sub-clusters or segments of approximately equal population size c) the random selection of one segment d) an interview with all households in the selected segment e) the selection of one child 6 to59 months old from each surveyed household. The survey was initially intended to determine the extent of *Plasmodium *infection in Malawi. Therefore, children under 6 months of age who are presumably protected from infection through the presence of maternal antibodies were excluded. There was a median number of 32 households per EA among 220 total EAs. The number of households per segment was indeterminate from the data. A total of 7,564 households were included in the final database (Table 1).

**Table 1:** Summary of household surveyed by sample locations and demographic characteristics, and of ITNs per household

	Households	
	**No**.	**%**

**All Districts**	7,564	100.0%

**District**		

Phalombe	930	12.3%

Blantyre	520	6.9%

Chiradzulu	736	9.7%

Mwanza	1,052	13.9%

Lilongwe	792	10.5%

Rumphi	1,162	15.4%

Nkhotakhota	1,189	15.7%

Karonga	1,181	15.6%

**SES Quintile**		

1 (lowest)	1,758	23.2%

2	1,661	22.0%

3	1,513	20.0%

4	1,391	18.4%

5 (highest)	1,241	16.4%

**Gender (Male)**	3,839	50.7%

**Age (Avg. months)**	25.12	--

**No. Children (per HH)**	1.26	--

**No. People (per HH)**	4.26	

**ITNs (Avg. per HH)**	1.35	--

**ITNs (Avg. per Child)**	0.31	--

### Data collection and definitions

Trained interviewers administered structured questionnaires to consenting household heads and/or parents/guardians of selected households to gather information on household ITN possession and use by children six to 59 months old, SES of the household, house construction, and other potential risk factors. The questionnaire was originally developed in English and translated into two main languages spoken in the selected Districts (Chichewa and Chitumbuka). Latitude and longitude of the house was taken using a GPS unit. Data were recorded electronically using a PDA and downloaded every evening of the survey onto a secure digital card, then later added to a master Microsoft Access database.

A household was defined as a family comprised of a male or female head, his or her husband or wife, as well as children and immediate family members who shared income. An ITN was defined as any bed net, regardless of treatment status, since for more than a decade treated nets only have been distributed in Malawi. Because the objective of this study was to evaluate health care access rather than net treatment practices, no attempt was made to distinguish between treated nets, time since retreatment, or long-lasting insecticidal nets (LLINs).

### Health facility data

**Figure 1:**
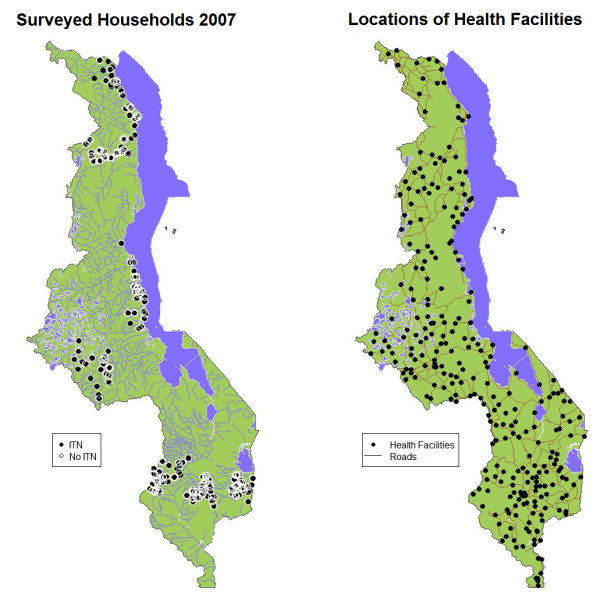
**Maps of Malawi showing (**a**) locations of households in the Northern, Central and Southern Regions surveyed during the 2007 sampling period and (**b**) Health facilities and major roads throughout the country**.

All HFs in Malawi, both public and private, were comprehensively surveyed in 2000 by the Japan International Cooperation Agency (JICA). The geographic coordinates for each HF, in addition to facility type, possession, funding source, type of road leading to the facility and road condition, were all recorded [33]. For the present analysis, only Malawi MoH hospitals, clinics, maternity clinics and dispensaries, and hospitals operated by religious groups (e.g. CHAM facilities) were considered (Figure 1). Although a small number of surveyed households of sufficient material means may have patronized private facilities, free net distribution and MoH health policies emanate from public HFs run by the MoH and cooperating religious organizations. Thus, as private clinics operate independently of MoH influence and policies with regard to malaria treatment and prevention, private clinics were excluded from this analysis.

### Distance to health facility measurement

Lacking individual data on travel methods, routes and travel times, distance to the nearest HF from each surveyed household was calculated using simple Euclidean distance calculated in ArcGIS 10.0. A more complex distance estimate with various assumptions about road use produced highly correlated values (data not shown). The HF located most proximal to each household may not always be the facility that households most consistently utilize. Data on preferred facilities, health seeking behaviours and health services were not available. In spite of this limitation, the facility of any type that was located closest to the surveyed household, was used assuming that the closest facility stands as an appropriate proxy for access to health services generally. Four types of HFs were considered: maternity/dispensaries, district hospitals, religious hospitals and primary health centres.

### Statistical methods

Associations between distance to nearest HF and ITN possession or use in the previous 24 h were explored. First, median distances to the nearest HF for the both ITN possession and use were compared using Wilcoxon tests of difference in medians. To determine trends in ITN possession and use in relation to increasing distance to HF, the complete set of distances to nearest HFs from households was broken into 10 equal and ordered groups (deciles). Next, percentages of sampled children in each category were calculated within each decile. Visual representations of the percentages of households within each decile were produced and a non-parametric loess curve to demonstrate trend was fit

Logistic regression was used to statistically test the relationship between increasing distance and ITN possession and ITN use. Linear and quadratic terms for distance to nearest health facility were included, suspecting that the relationship of distance to outcomes would not be linear. Tests for statistical significance in both bivariate and multivariate models were performed. Factors such as SES, gender, age and the ratio of nets to children in the household might confound the relationship of distance to outcomes, motivating a multivariate model. To account for possible contributions of surrounding households to the outcome of interest, a spatial covariate representing the percentage of the nearest five sampled children who reportedly possessed or used ITNs was included.

The univariate (1) and multivariate (2) models were:

(1) $$\log\;\text{it}\;\left(\pi \right)\;=\;\beta_{0}\;+\;\beta_{1}\;\left(\text{Distance}\;\text{to}\;\text{HF}\right)\;+\;\beta_{2}\;\left(\text{Distance}\;\text{to}\;\text{H}{\text{F}}^{2}\right)$$

(2) $$\begin{array}{c} \log\;\text{it}\;\left(\pi \right)\;=\;\beta_{0}\;+\;\beta_{1}\;\left(\text{Distance}\;\text{to}\;\text{HF}\right)\;+\;\beta_{2}\;\left(\text{Distance}\;\text{to}\;\text{H}{\text{F}}^{2}\right)+\beta_{3}\text{Age}+\\ \beta_{4}\text{Gender}+\beta_{5}\text{Net}\;\text{Ratio}+\beta_{6}\text{SES}\;\text{quintile}+\beta_{7}\;\left(\%\;\text{of}\;\text{surrounding}\;5\;\text{neighbors}\right) \end{array}$$

Associations between the type of HF nearest the household and net, ITN possession, and ITN use were evaluated. Since differing levels of ITN distribution occur at the different facilities, patterns of association with net possession, ITN possession and ITN use were tested using logistic regression and a categorical predictor of the four health facility types of interest: maternity/dispensaries, district hospitals, religious hospitals and primary health centres. All statistical analyses were performed in R, version 10.1.1 (CRAN.org).

## Results

Overall, 75.8% of surveyed households reported owning an ITN. Of all households surveyed, 69.5% stated that the child chosen for the survey slept under a net the previous night. ITN possession and ITN use varied among districts, with Karonga reporting the highest levels of use (88.1%) and possession (85.3%). Households in Mwanza District had the lowest levels of ITN possession (76.0%), while Chiradzulu had the lowest level of ITN use by children within the previous 24 h (45.1%) (Table 2).

**Table 2:** Percentages of households reporting possession of at least one ITN and use of ITNs by the surveyed child in the previous 24 hours by district and wealth quintile

	Possesses ≥ 1 ITN		Child Slept Under ITN	
	**No**.	**%**	**No**.	**%**

**All Districts**	5738	75.8%	5195	69.5%

**District**				

Phalombe	768	82.6%	688	74.5%

Blantyre	338	65.0%	281	54.2%

Chiradzulu	492	66.8%	332	45.1%

Mwanza	800	76.0%	724	68.8%

Lilongwe	573	72.3%	499	63.6%

Rumphi	840	72.2%	803	69.7%

Nkhotakhota	919	77.2%	868	73.1%

Karonga	1008	85.3%	1000	88.1%

**SES Quintile**				

1 (lowest)	1239	70.4%	1127	64.1%

2	1249	75.2%	1144	68.8%

3	1188	78.5%	1099	72.6%

4	1060	76.2%	948	68.1%

5 (highest)	1002	80.7%	941	75.8%

ITN possession and use followed similar patterns among SES groups. Households in the lowest quintile of material wealth reported the lowest levels of ITN possession and use, while possession and use were highest among the wealthiest of households. The middle three SES quintiles did not vary appreciably from one another

### Distance, possession and ITN use

Wilcoxon tests statistically confirmed differences between distances to nearest HF and ITN possession and use. Households that did not possess ITNs and which reported that children did not sleep under one the previous night were located further away than households that possessed and used ITNs. Households that reported possessing an ITN were located on average 3.87 km from the nearest HF, whereas households that did not possess one were located 4.65 km away. Households reporting that the surveyed child slept under an ITN the previous night were located 3.85 km away from the nearest facility, while households that reported that the child did not sleep under an ITN were more distant, being located 4.24 km away. Both differences were statistically significant (*p *< 0.0001).

**Figure 2:**
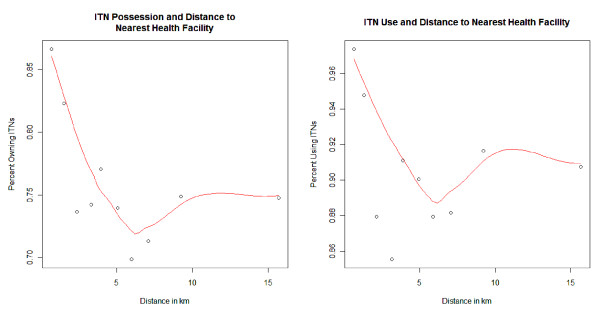
**Possession of any type of net and possession of ITNs by quantile of household distance to nearest health facility (left) along with use of any type of net in the previous 24 h by quantile of household distant to nearest health facility for 7,564 households**. Lines represent loess (locally weighted scatterplot smoothing) curve fits to illustrate trends.

Household possession and use of ITNs in the previous 24 h followed similar trends with increasing distance. Both possession and use were highest in households most proximal to health services and lowest among those farthest away from HFs. The trend in declining ITNs with distance to the nearest HF was not linear, rather dropped sharply for households within ~5-7 km, then leveled off for the remaining households further away (Figure 2). Patterns of ITN use given possession of at least one ITN showed the same distance-decay pattern as that for possession only. "Hockey stick" regression was used to determine an optimal breakpoint in the associative trend of distance on ITN possession and use, noting that the trend of decay in the loess interpolation appears to change strikingly at ~5-7 km. There was a breakpoint in distance and ITN possession at 5.2 km, where possession stops declining with distance and becomes constant. There was also a breakpoint of 6.4 km for ITN use given possession. The increase in use for very remote households was worth noting. This study only includes known HFs and does not take into account the locations of community-based health services which could be driving this phenomenon.

Logistic regression models confirmed statistical significance of distance to nearest HF for ITN possession and ITN use given possession in the previous 24 h in both bivariate models and multivariate models with possible confounding covariates. Linear and quadratic terms for distance were significant in all models. While use of ITNs by neighboring households was the most important predictor of both net possession and net use given possession among surveyed children, the effect of distance upon net use remained unchanged, both in magnitude of effect and pattern (Table 3). Interestingly, SES was not significantly associated with ITN possession or use. However, age was significantly associated with ITN use, implying that households may prioritize ITN use for very young children who sleep alongside mothers.

**Table 3:** Results of logistic regression models of possession of any kind of net, ITN possession and reported ITN use in the previous 24 hours

	Net Possession		Slept Under ITN	
	**OR**	**p-value**	**OR**	**p-value**

**Intercept**	5.23 (4.56, 6)	< 0.0001**	17.58 (15.33, 20.16)	< 0.0001**

**Distance**	0.82 (0.78, 0.87)	< 0.0001**	0.75 (0.72, 0.79)	< 0.0001**

**Distance^2^**	1.01 (1.01, 1.02)	< 0.0001**	1.02 (1.02, 1.03)	< 0.0001**

**Intercept**	0.36 (0.26, 0.5)	< .0001**	2.17 (1.28, 3.69)	0.004*

**Distance**	0.92 (0.87, 0.97)	0.002*	0.87 (0.78, 0.96)	< 0.005*

**Distance^2^**	1.01 (1, 1.01)	< 0.015*	1.01 (1, 1.02)	< 0.002*

**Age**	1 (1, 1.01)	0.531	0.98 (0.97, 0.99)	< 0.0001**

**Gender**	0.97 (0.87, 1.09)	0.662	1.02 (0.85, 1.24)	0.806

**SES**	1 (0.96, 1.05)	0.88	0.95 (0.88, 1.02)	0.157

**Neighbors**	28.83 (23.28, 35.7)	< 0.0001**	33.07 (24.05, 45.46)	< 0.0001**

**Net Ratio**		NA	1.36 (0.88, 2.1)	0.173

Both bivariate and multivariate are included

* = p < .01, ** = p < .0001

The covariate representing ITN possession and use among proximal neighbors was intended to account for spatial dependencies in the data which could negatively affect the estimation process. Households which are located in communities where ITN possession is high are, of course, more likely to own nets themselves. In terms of possession, for example, community ITN coverage will be related to distance to the nearest health services as a point of distribution and to the outcome of household ownership. Thus, this variable was introduced in the multivariate models as a potential confounder to be controlled for. The very high contribution of proximal ITN possession on household possession in the multivariate logistic model confirms this assumption (OR 28.83). However, as households do not receive nets from one another, further exploration of this covariate was deemed unnecessary although the authors recognize that neighbors may influence one another in procuring ITNs from health facilities. Conversely, the neighbor covariate for ITN use among possessing households was of interest since household behavior may be influenced by the behavior of one's neighbors. The odds of ITN use among households which were located in communities where there is universal coverage among proximal neighbors were 33 times higher than households located in communities where no people use ITNs. An assessment of spatial autocorrelation in ITN use produced a Moran's I statistic of 0.30 (*p *< 0.001), indicating that households proximal to one another were more likely to exhibit similar ITN use behaviours.

Despite the presence of the neighbor variable in models of both ITN possession and ITN use, the variable for distance to nearest health services remained significant. The association of distance to health services on ITN possession and use appears small, but not so small when considering the cumulative effects of distance. The OR for ownership of ITNs for households 5 km away from the nearest health services was 0.45 in the bivariate model and 0.84 in the multivariate model. Similarly, the OR for use given possession for households located 5 km from the nearest HF was 0.38 for the bivariate model and 0.63 for the multivariate model.

### Health facility type

Households that were located closest to a district hospital had elevated odds (OR 2.64 (2.19, 3.19)) of possessing at least one ITN compared with those located close to dispensary/maternity clinics. However, ITN possession in households located near religious hospitals (OR 1.13 (0.83, 1.53)) and primary health centres (OR 1.07 (0.91, 1.24)) was not significantly different from that in households close to dispensary/maternity clinics.

The odds of sleeping under an ITN were significantly higher among households that were closest to district hospitals (OR 2.67 (1.93, 3.69)) than those closest to dispensary/maternity clinics. Religious hospitals (OR 1.14 (0.68, 1.89)) and primary health centres (OR 1.08 (0.84, 1.4)) did not differ significantly from dispensary/maternity clinics in ITN use (Table 4).

**Table 4:** Results of logistic regression modelling ITN possession and reported ITN use within the previous 24 hours by type of nearest health facility

	Facility Type	OR	p-value
**ITN Possession**	**Intercept**	**2.77 (2.60, 2.94)**	**< 0.0001**
	
	**Dispensary/Maternity**		
	
	**District Hospital**	**2.64 (2.19, 3.19)**	**< 0.0001**
	
	**Religious Hospital**	1.13 (0.83, 1.53)	0.43
	
	**Primary Health Centre**	1.07 (0.91, 1.24)	0.42

**Slept Under ITN**	**Intercept**	**8.33 (7.52, 9.23)**	**< 0.0001**
	
	**Dispensary/Maternity**		
	
	**District Hospital**	**2.67 (1.93, 3.69)**	**< 0.0001**
	
	**Religious Hospital**	1.14 (0.68, 1.89)	0.62
	
	**Primary Health Centre**	1.08 (0.84, 1.40)	0.55

Statistically significant health facility types are noted in bold

## Conclusions

Using simple methodologies, the authors have shown that distance to health services is associated with ITN possession. Interestingly, this analysis uncovered the same relation with reported household use of any type of nets, even among those households who possess at least one. This finding provides further evidence to suggest that health services may play an important role in providing not only material resources, but also promoting use of health interventions within the home. Although data on maternal attitudes toward local health workers and preferred sources of health information were not available in this study, the results support the inference that regular contact between citizens and health workers or health intervention distribution sites help promote and reinforce beneficial household health behaviours.

In Malawi, present strategies of ITN distribution centre on antenatal and under-five clinics. Given the results of this study, which suggest that possession of ITNs decreases with increasing distance from health facilities, there may be a need to enhance community-centred ITN distribution models, to achieve the same level of coverage as existing community-based vaccination strategies. ITNs could be distributed widely within communities, utilizing present community health workers to deliver them directly to households. Information on appropriate use should then be disseminated directly to caregivers, reinforcing proper protective behaviors. Other studies have shown that community based malaria education programs result in more consistent patterns of households ITN use [34]. An emphasis on a community-based model over facility-based distribution strategies could address the problem of low ITN possession particularly among communities distant from health services, but could also serve to encourage consistent levels of use within households in isolated communities that have little access to malaria educational programmes.

The mechanisms of household-level decision-making as they relate to HF access are largely unknown. More frequent trips to HFs might result in more opportunities for health messages to reach households, reinforcing pro-active efforts to protect the health of family members and increasing awareness of the causes and prevention of malaria. This study lacked a quantified measure of education of female household heads, preventing formal examination of this possibility. Other studies have indicated that knowledge and perceptions of malaria sources varied among education levels [23], with better educated mothers having greater malaria knowledge than those who were less educated. In addition, Dyke, et al [35] demonstrated in Nigeria that higher levels of education are not only associated with malaria knowledge, but also with actual ITN use.

Health services tend to be located near markets, schools and other important areas of infrastructure. Thus, households near HFs are also likely to be of greater SES through participation in market economic activities, better educational opportunities and greater chances for employment. Residents living further away from health services, and thus from market centres, may tend to be less educated, less likely to participate in cash-based economic activities, and less prone to taking advantage of health services and interventions. However, because education level and SES are intertwined, the lack of significance of SES (wealth quintiles) in the regression model for household ITN use suggests that education plays less of a role in ITN use than health messages and access to services than might be assumed. While the insignificance of SES in the multivariate possession model may be the result of ITN distribution programmes that specifically target low SES households, the lack of significance in a model of ITN use given possession suggests the need for further exploration of determinants of ITN use beyond material wealth.

The authors recognize that there may have been systematic over-reporting of ITN use or variability in reports of ITN use among SES groups, for example. Respondents may have been concerned about self-implicating themselves if they believed that they neglected to protect their children from disease. Similarly, no clear attempt was made to disentangle actual practices from reported habits through structured verification of responses and the data prevented validation of responses of ITN use. However, misreporting of ITN use was probably unrelated to distance to nearest HF, suggesting no systematic bias that would influence statistical estimates of ITN use and health facility proximity associations. While absolute percentages of ITN use may be overestimated in the present survey, the reported patterns with proximity to health services are valid and representative.

Equitable distribution of ITNs with strategies that maximize coverage of high risk areas should be of uppermost priority among health officials. The authors recommend that health workers take proactive steps to help communities that are remotely situated (particularly those beyond 5 km) from health services. Although nothing in these results allows one to test the hypothesis that direct, community-based health initiatives reinforce health behaviors, future research should attempt to measure the public health impact of community workers. Studies that assess community attitudes toward those workers could direct policy makers to upgrade current strategies which could, in turn, help improve the level of trust in the information and services that they deliver.

Clearly, though, community based malaria programmes should not be limited to ITNs, but should include other methods such as indoor residual spraying (IRS) and home-based treatment methods. Promoting a comprehensive approach in the fight against malaria should be of the utmost importance to both researchers and policy makers [36]. IRS initiatives may obviate the need for regular, nightly use of ITNs and home treatment strategies for isolated areas may mitigate transmission levels. To this end, future research efforts should include other intervention methods, access to which may also be associated with distance to health services. Future research could utilize the methods presented here, to assess the potential role that access to health services can play in an interdependent system of interventions and work to target underserved geographic areas of significant risk for malaria. It is possible that within this system of interventions, some may be more effective than others and the correct 'recipe' for a sustainable strategy balanced with logistical costs may be geographically dependent on access to health services.

## Competing interests

The authors declare that they have no competing interests.

## Authors' contributions

PSL performed the data analysis, reviewed the literature, interpreted the statistical results, and drafted the manuscript. DPM participated in the study design, supervised data collection, and reviewed the manuscript. CHC participated in the study design and reviewed the manuscript. MLW assisted with the analysis plan, helped interpret the results and critically revised the manuscript. All authors read and approved the final manuscript.
